# Homeopathy for mental fatigue: lessons from a randomized, triple blind, placebo-controlled cross-over clinical trial

**DOI:** 10.1186/1472-6882-12-167

**Published:** 2012-10-01

**Authors:** Michael Emmans Dean, Raj Karsandas, J Martin Bland, Debbie Gooch, Hugh MacPherson

**Affiliations:** 1 Dept. of Health Sciences, University of York, York YO10 5DD, UK; 2 Dept. of Psychology, University of York, York YO10 5DD, UK

**Keywords:** Crossover study design, Mental fatigue, Kali phos 6x, Stroop test

## Abstract

**Background:**

Difficulty in controlling attention can lead to mental fatigue in the healthy population. We identified one trial reporting a benefit in patients’ attention using a homeopathic formula preparation. One component of the preparation was potassium phosphate, widely available off the shelf as *Kali phos* 6x for cognitive problems. The aim of this exploratory trial was to assess the effectiveness of *Kali phos* 6x for attention problems associated with mental fatigue.

**Methods:**

We recruited student and staff volunteers (University of York) with self-reported mental fatigue, excluding any using homeopathy or prescribed stimulants, or with a diagnosis of chronic fatigue syndrome. In a triple blind, cross-over, placebo-controlled clinical trial, 86 volunteers were randomized to receive *Kali phos* 6x or identical placebo 10 minutes before taking a psychological test of attention (Stroop Colour-Word Test). One week later they were crossed over and took the other preparation before repeating the test.

**Results:**

We found no evidence of a treatment effect in a comparison of *Kali phos* 6x with placebo (*Kali phos* minus placebo = −1.1 (95% CI −3.0 to 0.9, P = 0.3) Stroop score units, Cohen effect size = −0.17) even when allowing for a weak period effect with accuracy scores in the second period being higher than those in the first (P = 0.05). We observed a ceiling effect in the Stroop test which undermined our ability to interpret this result.

**Conclusions:**

*Kali phos* 6x was not found to be effective in reducing mental fatigue. A ceiling effect in our primary outcome measure meant that we could not rule out a type II error. Thorough piloting of an adequate outcome measure could have led to an unequivocal result.

**Current Controlled Trials:**

ISRCTN16521161

## Background

This research was part of a programme evaluating homeopathy for attention-deficit/hyperactivity disorder or hyperkinetic disorder (ADHD and HKD). We have published a Cochrane Library review of homeopathy for ADHD and HKD [1]. The review identified four randomized or quasi-randomized placebo-controlled trials [2-5] that used behavioural rating scales, three of which also used subscales of the Conners test [6]. Of the four studies, three used individualized ‘classical’ homeopathy and substantial differences in prescribing methods between trials and conflicting results make this group of studies hard to interpret [7,8]. A formula ‘complex’ preparation was tested in the fourth study [4] and appeared to benefit patients’ attention in the Children’s Checking Task [6]. However, there has been no independent replication, or further research into the medication. The clinical effects of its individual components (homeopathic dilutions of potassium phosphate and selenium), are only known from anecdotal reports in the homeopathic literature.

Inattention is a problem experienced by many people, especially those with ADHD in whom it is one of three core symptoms that can lead to serious psychological, social and educational/occupational impairment [9]. It has been proposed that control of attention, impulses and thoughts is based on a shared mechanism and that mental fatigue sets in after extensive use of this mechanism, which may then lead to impaired cognitive function [10].

Potassium phosphate is traditionally indicated in standard homeopathic sources for cognitive problems including inattention and concentration difficulties [11]. It is commonly available off the shelf (official nomenclature *Kalium phosphoricum*, abbrev. *Kali phos*) with similar indications, but there has been no independent research into its efficacy. As we had encountered difficulties in gaining ethics approval and support from parents and care givers for a study with young people diagnosed with ADHD, we therefore designed an exploratory clinical trial with an efficient cross-over design to evaluate *Kali phos* in treating the specific symptom of mental fatigue in healthy adult volunteers. In this paper we present the results of the current trial and some of the difficulties and problems with the methodology.

## Methods

### Objectives

To assess the effectiveness of homeopathic potassium phosphate (*Kali phos* 6x), an off-the-shelf preparation, for attention problems associated with mental fatigue in healthy adults.

### Participants

We recruited 86 adult volunteers via online advertisement and internal circulation of emails within the University of York. To be eligible for the study participants had to self-report difficulties in sustaining attention or be experiencing mental fatigue. Additionally they had to be able to communicate in English and consent to avoiding the use of self prescribed stimulants, such as caffeine and energy drinks, on the day of each test. We excluded those who were currently using a homeopathic preparation for any condition, people who were currently using prescribed stimulant medication such as those used for ADHD and people diagnosed with chronic fatigue syndrome or ME. Those eligible were offered information leaflets and an explanation of the study by the CI (MED). All participants gave written consent to participate.

### Interventions

The homeopathic preparation was a single dose of 0.6 g of lactose powder medicated with *Kalium phosphoricum* 6x (a decimal dilution equivalent to 1 part in 1 000 000, potentised by serial agitation) in 90% ethanol/water solution. The placebo was a single dose of 0.6 g of lactose powder treated with unmedicated 90% ethanol/water solution. There was no noticeable difference in taste or appearance between the two preparations. Both preparations were supplied by the Helios Pharmacy, London, who coded the batches A and B so that nobody at the trial centre was aware which powder was placebo and which *Kali phos*. The identity of powders A and B was not revealed by the pharmacy until after completion of the analysis. The pharmacy also supplied the manufacturer’s data sheet for the lactose used in both preparations.

### Study design

The study design was a triple-blinded, placebo-controlled cross-over trial with two arms in which participants were randomly allocated to receive either a homeopathic or placebo preparation in period 1 and vice versa in the period 2.

In both periods the participants completed the 4-question mental fatigue sub-scale of the Chalder Fatigue Questionnaire, giving an integer score between 0 and 4 [12]. They subsequently took a single dose of one of the randomly allocated preparations. They familiarized themselves with the software in trial runs of the psychological test of attention. They then performed the test, approximately ten minutes after taking either the homeopathic or placebo preparation. Each participant repeated the procedure at the same time of day, seven days later, those who received *Kali phos* in period 1 receiving the placebo preparation and vice versa. After completing the period 2 test, participants were asked whether they thought they had just taken *Kali phos* or placebo.

### Outcomes

A review by Swanson et al. concluded that of three domains of assessing cognitive deficits, conflict resolution tasks (CRT) were the best at distinguishing children in ADHD diagnosed groups from control groups [13]. Of these CRTs the Stroop Colour-Word Test has been identified as one of the most sensitive for testing ADHD-specific deficits [14]. Therefore our primary outcome measure was accuracy score on the Stroop Colour-Word Test [15].

The Stroop task involved participants being shown a colour word (e.g. ‘red’) which was coloured either congruently (e.g. the word ‘red’ was coloured red) or incongruently (e.g. the word ‘red’ was coloured green). Participants were required to respond to either the word or the colour of the word by pressing corresponding keys on the computer keyboard (1 = red, 2 = blue, 3 = green). Participants completed 2 practice blocks each containing 9 trials (6 incongruent and 3 congruent). In the first block participants were instructed to respond to the word and in the second block participants were instructed to respond to the colour of the word. Feedback on performance accuracy was provided during these practice blocks. Six test blocks, each containing 27 trials (18 incongruent trials and 9 congruent trials) were then presented. For the first, third and fifth blocks participants responded to the word whereas for the second, fourth and sixth blocks participants responded to the colour of the word. A recovery period of 30 seconds in between each test block was included so that the task was suitable for a linked functional magnetic resonance imaging investigation (this follow-on project was not undertaken). The dependent variable was participants’ accuracy score on the incongruent trials (maximum = 108). The test lasted approximately 18 minutes. The measure was adapted for computer presentation using EPrime® software [16] and was used in both periods. We originally aimed to measure the speed of response as a secondary outcome, but the software was programmed with a 3-second delay before the response screen appeared and therefore this could not be reliably measured.

### Sample size

We wanted to be able to detect a fairly small difference in the Stroop score and therefore decided to design the study to detect an effect size of 0.3 standard deviations with power 0.90 and using a significance level of 0.05. For a cross-over trial, we would need the standard deviation of differences between repeated measures at a similar time gap as we proposed for the trial, or the correlation coefficient between such pairs of observations. We could find no pre-existing data allowing us to estimate this correlation, and a pilot study to estimate this would have been almost as time-consuming to carry out as the present study. We therefore arbitrarily set the correlation between the accuracy scores in the first and second periods to be 0.80, believing this would be a reasonable value for a continuous measurement recorded with only seven days between measurements. We estimated that 86 volunteers would be required to give the desired power.

### Allocation to treatment order

Allocation was random using our software Clinstat [17] to allocate 86 participants into equal groups in blocks of random sizes 4, 6, 8, or 10. For each participant, the folded papers containing powders A and B were put by JMB into clear plastic envelopes and labelled with the participant number and either “first” or “second”. The completed packages were passed to MED who administered them to the participants, all of whom were fully blind to the allocation.

### Statistical analysis

Statistical analysis followed the method of Hills and Armitage [18] using the description of Altman [19]. We have followed Senn by not testing the interaction between treatment and period [20]. As the assumptions of the two sample t methods used were not well met by the data, Mann Whitney U tests were used to confirm the analyses.

### Ethical approval

Ethical approval was given by the University of York Health Sciences Research Governance Committee on December 12, 2006. The trial registration number is ISRCTN16521161.

## Results

### Participants

**Figure 1:**
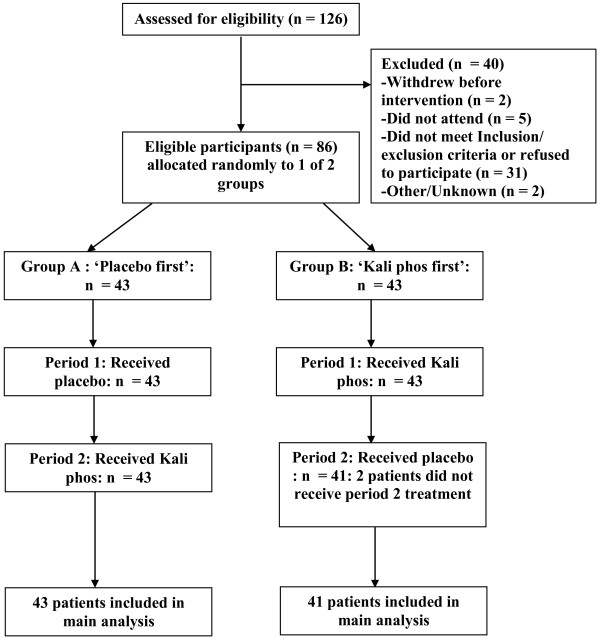
Participants’ flow chart.

In 2007, 86 participants who were eligible for the study were randomly allocated to two groups, group A to receive placebo first and *Kali phos* second and vice versa for group B after a one week ‘washout’ period. 84 participants completed both periods of the study (Figure 1) and two participants, both in Group B, were unable to attend period 2. They were treated as missing completely at random and not included in the analysis.

Table 1 shows the baseline demographic characteristics and pre-test data for both groups. The majority of participants were female and the majority were students. The pre-test Chalder Mental Fatigue Score fell significantly between period 1 and period 2 (P = 0.0004, sign test).

**Table 1:** Baseline demographics and pre-test data

**Characteristic**	**Placebo first:**	**Kali phos first**
	** n = 43**	** n = 43**
Mean age ± SD	33.7 (13.3)	33.44 (13.6)
Female:Male ratio	34:9	31:12
Student:staff ratio	23:20	18:25
Mean (SD) pre-test Mental Fatigue Score* for period 1	2.8 (1.1)	2.7 (1.0)
Mean (SD) pre-test Mental Fatigue Score* for period 2	2.3 (1.3)	2.1 (1.2)
* Based on the Chalder Fatigue Questionnaire		

### Accuracy scores

**Figure 2:**
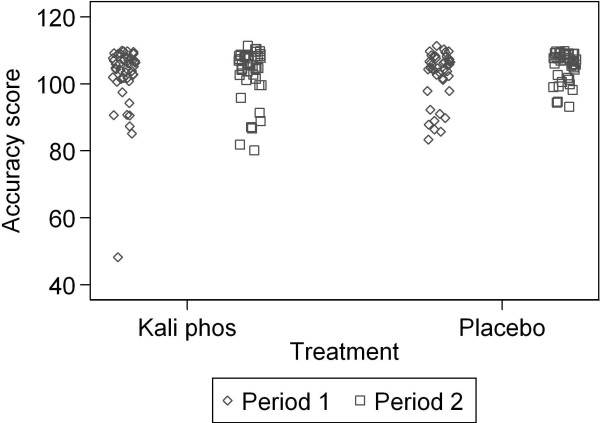
Accuracy scores for both groups in both periods.

Table 2 shows the accuracy scores for Groups A and B over both periods of the trial. The data are shown graphically in Figure 2. Several things can be seen in these data. The distribution of score is highly negatively skew, with a long tail of lower values. There is a clear ceiling effect, with many participants achieving the maximum score of 108 in both periods of the trial. There is one apparent outlier in *Kali phos* period 1. There is little to suggest a treatment effect, but there may be a period effect, with period 1 have lower scores than period 2.

**Table 2:** Accuracy scores on the Stroop Colour-Word Test for 86 participants in groups A and B, for periods 1 and 2

**Placebo first**				**Kali phos first**			
**Placebo**	**Kali phos**	**Placebo**	**Kali phos**	**Kali phos**	**Placebo**	**Kali phos**	**Placebo**
84	108	106	104	50	101	105	107
85	108	106	107	86	99	105	108
88	82	106	107	89	106	106	96
88	89	106	107	91	102	106	108
88	107	106	108	92	100	106	108
91	104	106	108	93	106	106	108
92	107	106	108	93		106	.
93	89	107	100	97	106	107	105
98	89	107	104	99	106	107	106
98	107	107	105	101	103	107	106
101	80	107	107	102	95	107	106
101	90	107	107	102	99	107	107
101	99	107	108	102	101	107	107
103	98	107	108	102	101	107	108
103	106	108	94	102	106	108	107
103	107	108	104	102	108	108	107
104	107	108	106	102	108	108	108
104	108	108	108	103	105	108	108
105	106	108	108	103	108	108	108
105	107	108	108	104	90	108	108
105	108	108	108	105	104	108	108
106	100			105	107		

The average correlation between the first and second scores across the two orders was 0.32. This was much smaller than the 0.80 postulated in the design of the experiment. First we asked whether there was evidence for a period effect, i.e. are the Stroop accuracy scores in the first period the same as in the second? For example, there might be a learning effect, with accuracy increasing with repetition of the test. If there were no period effect, we would expect the differences between the treatments to be the same in the two periods. The period effect, second period minus first period, is estimated by the treatment difference for period 2 minus the treatment difference for period 1. This is 3.9. We can estimate a 95% confidence for this using the two sample t method, giving 0.0 to 7.8. There is weak evidence of a period effect, P = 0.05, mean accuracy scores increasing from period 1 to period 2. Although the two sample t test is robust to departures from the assumption of a Normal distribution, particularly when groups sizes are very similar, the data clearly do depart from this (Table 2 and Figure 2). We therefore also tested the period effect using the Mann Whitney *U* test. This also provided rather weak evidence of a period effect, P = 0.05.

We estimated the treatment difference allowing for a possible period effect from the difference between the mean differences between periods for those who receiving placebo first and those who received *Kali phos* first. The estimate of the effect, *Kali phos* minus placebo, = −1.1 (95% CI −3.0 to 0.9, P = 0.3) Stroop score units. There is no evidence for a treatment effect. The non-parametric equivalent is a Mann Whitney *U* test of the difference, period 1 minus period 2, between the two orders. Again there is no evidence for a treatment effect, P = 0.3. The two analyses give very similar results.

The standard deviation of the first Stroop scores within treatment groups is 8.6. If we exclude the outlier, we get 6.5. If we take this as a reasonable estimate of the baseline standard deviation, we can use it to estimate the treatment effect in standard deviations. Dividing the difference in score for *Kali phos* minus placebo by 6.5 gives −0.17 (95% CI −0.46 to 0.13). Hence the estimated treatment effect is much smaller than the 0.3 which this trial was designed to detect and is in the opposite direction, and 0.3 is not included within the confidence interval and so would not be consistent with the data.

### Mental fatigue

The change in the pre-test mental fatigue score from first to second period was highly significant. Participants were selected to have self-reported fatigue, but this was not always reflected in the fatigue score. Two participants had a fatigue score = 0, no fatigue, at the first measurement. They were not screened out on the basis of this score. We asked whether the fall in mean fatigue score could be a treatment effect? There was no evidence of this. The mean fatigue scores at the second period were 2.3 for participants receiving placebo first and 2.1 for participants receiving Kali phos first (P = 0.4, Mann Whitney *U* test). The corresponding mean falls in fatigue score were 0.5 and 0.6 (P = 0.5).

We then asked whether the change in pre-test mental fatigue score could explain the period effect. We did this by analysis of covariance, with treatment, order, and subject as fixed categorical factors and fatigue score as a quantitative covariate. The estimated treatment effect, *Kali phos* minus placebo, was −1.2 (95% CI −3.1 to 0.8, P = 0.2), almost identical to the unadjusted analysis. The estimated period effect, second minus first, was 2.9 (95% CI 0.8 to 5.0, P = 0.007), smaller than in the unadjusted analysis but more highly significant. Hence, although there is evidence that fatigue score did explain some of the increase in score from the first to the second period, it did not mask any treatment effect.

### Participants’ identification of treatment

Participants were asked at the second visit whether they could identify which treatment they had just received, *Kali phos* or placebo. Of the 84 participants at period 2, 23 guessed *Kali phos*, 39 guessed placebo, and 22 did not express an opinion. Of the 62 willing to guess, 29 were correct and 33 were incorrect. There is no evidence that guesses were other than random (P = 0.7, sign test comparing proportion correct with proportion incorrect). Of those willing to guess, *Kali phos* was the choice of 10/30 who actually received *Kali phos* and 13/32 who actually received placebo (P = 0.6, chi-squared test).

### Potassium content of homeoepathic and placebo preparations

The manufacturer’s data sheet for the lactose powder identified numerous trace elements including potassium (<0.01%). We performed our own chemical analysis using an atomic absorption spectrophotometer in the Chemistry Department at the University of York which showed a potassium content of approximately one part per million in both *Kali phos* and placebo preparations.

## Discussion

### Synopsis of key findings

In a comparison of a homeopathic preparation (*Kali phos* 6x) with placebo, in a two period triple blinded cross over study, we found no evidence of a treatment effect. There was weak evidence of a period effect where accuracy scores in the second period were higher than those in the first across both treatment groups. This period effect was not explained by a decrease in pre-test mental fatigue scores from period 1 to period 2. This decrease in mental fatigue scores may be attributable to regression towards the mean. Chemical analysis showed that the (potentized) homeopathic and placebo preparations both contained approximately 1 part per million of potassium. Trace elements are not believed to affect the difference between potentized and unpotentized substances, as it is the potentization that is considered to provide the mechanism associated with therapeutic benefit.

### Consideration of findings in respect to the literature

Our Cochrane review of homeopathy for attention deficit/hyperactivity disorder [1] found two trials which measured the specific domain of inattention through child completed tasks [4,5]. Although the trial of a formula preparation of homeopathic potassium phosphate and selenium reported some benefit in attention using the Children’s Checking Task [6], when percentage data were converted into raw scores and reanalysed there was no significant difference between homeopathy and placebo [1]. A pooled estimate of the two trials also indicated no evidence of the effectiveness of homeopathy for ADHD [1], consistent with the findings of the current study.

### Limitations of study

A clear ceiling effect, as demonstrated in Figure 2, shows that a large number of accuracy scores were at or close to 108, the maximum score achievable. It meant that participants who achieved close to the maximum score in period 1 had little room for improvement in period 2. This reduced possible difference between mean scores, but it also reduced the variance of measurements between participants, so may not have greatly affected the estimated effect size. The ceiling effect also reduced the correlation between repeated observations. The original sample size calculation postulated a correlation of 0.8 between scores in the first and second periods. The actual correlation was only 0.3. This must have led to a reduction in power to detect a treatment difference. The confidence interval for the treatment effect size remained quite narrow and clearly excluded the difference we set out to detect, but it remains possible that a more sensitive measurement might produce a different result.

Despite the lack of a positive finding, we support authors such as Dwan et al [21]. who advocate publishing all studies in order to avoid publication bias in subsequent reviews and meta-analysis. In addition, such studies such as ours can highlight ways to improve the research.

The ceiling effect observed was a result of a limitation in our implementation of the outcome measure used, the Stroop Colour-Word Test. The test design included a recovery period of 30 seconds between each block because the test was intended to be used in a parallel neuroimaging study using functional magnetic resonance imaging. Additionally there was a 3 second gap before each response could be made. Both these factors meant that many participants found that the test was not sufficiently challenging. Preliminary assessment of the test was informally performed on a small number of volunteers, however a full pilot study was not. Pilot studies for cross-over trials are seldom worth conducting as they require almost as many participants and resources as the actual trial. As Senn notes [20], cross-over trials are carried out where the condition is not life-threatening and there is usually no reason why they should not be repeated with a different design. This is certainly the case here and if this question were thought worthy of further research another trial could be carried out using a more appropriate version of the Stroop test with no ceiling effect.

### Clinical and research implications

Several systematic reviews have outlined the weak evidence for the specific effect of homeopathic remedies over placebo [22,23]. They go on to highlight the lack of rigorously conducted randomized controlled trials that have been published. Such trials have been historically difficult to perform in the field of complementary and alternative medicine primarily because of the lack of funding and because of difficulties in recruiting adequate numbers of participants [24].

‘Homeopathy’ is an umbrella term that covers many different methods of diagnosis and prescription [7]. It has been argued that placebo-controlled trials are inappropriate in the evaluation of individualised ‘classical’ homeopathy, which requires therapist-intensive consultations and individualized prescriptions based on each patient’s symptom picture and unique characteristics [25]. However formulaic ‘clinical’ homeopathy is widely available as standardized treatments for predefined conditions or symptoms making it appropriate for rigorous randomized, placebo-controlled clinical trials, as in the current study.

## Conclusions

Although the current study failed to find any significant effect of the *Kali phos* 6x on mental fatigue, this may be attributable to the methodological flaws in the implementation of the outcome measure causing a ceiling effect, so a treatment effect cannot be ruled out. This study also has implications for further research in complementary and alternative medicine, providing an example of a resource-efficient cross-over design to evaluate a treatment for the temporary alleviation of a stable condition rather than a cure.

## Competing interests

The authors declare that they have no competing interests.

## Authors’ contributions

MED was the principal investigator and carried out the trial, JMB did the statistical design and randomization, including preparation of the concealed treatments, and carried out the statistical analysis, RK drafted the first version of the paper, DG prepared the Stroop test, HM was involved in the design of the study and interpretation of the results. All authors were in involved in drafting and approving the final paper.

## Pre-publication history

The pre-publication history for this paper can be accessed here:

http://www.biomedcentral.com/1472-6882/12/167/prepub
